# Successful treatment with positive airway pressure ventilation for tension pneumopericardium after pericardiocentesis in a neonate: a case report

**DOI:** 10.1186/s40981-020-00384-x

**Published:** 2020-10-07

**Authors:** Makiko Tani, Tomoyuki Kanazawa, Naohiro Shioji, Kazuyoshi Shimizu, Tatsuo Iwasaki, Hiroshi Morimatsu

**Affiliations:** 1 Department of Anesthesiology and Resuscitology, Graduate School of Medicine Dentistry and Pharmaceutical Sciences, Okayama University, 2-5-1, Shikata-cho, Kita-ku, Okayama, 700-8558 Japan; 2 Department of Anesthesiology and Resuscitology, Okayama University Hospital, 2-5-1, Shikata-cho, Kita-ku, Okayama, 700-8558 Japan

**Keywords:** Pneumopericardium, Pericardiocentesis, Recurrent nerve palsy, Pleural pressure, Positive pressure ventilation

## Abstract

**Background:**

Pneumopericardium in neonates is often associated with respiratory diseases, of which positive pressure ventilation (PPV) is an exacerbating factor. Here, we present a neonate case of pneumopericardium after cardiac surgery which was resolved after applying PPV.

**Case presentation:**

A 28-day-old neonate with left recurrent nerve palsy after aortic reconstruction for interrupted aortic arch developed pericardial effusion. Pericardiocentesis was performed under general anesthesia, and a drainage tube was left in the pericardium. After extubation, stridor gradually exacerbated, following hemodynamic deterioration. A chest X-ray demonstrated pneumopericardium. Upper airway stenosis due to recurrent nerve palsy developed excessive negative pleural pressure, and air was drawn into pericardium via the insertion site of the drainage tube. After tracheal intubation and applying PPV, the pneumopericardium improved.

**Conclusion:**

PPV does not always exacerbate pneumopericardium. In a patient with pericardial-atmosphere communication, increased inspiration effort can cause pneumopericardium, and PPV is a therapeutic option to alleviate the pneumopericardium.

## Background

Pneumopericardium is defined as the collection of air or gas in the pericardium [1]. Pneumopericardium is categorized into two types according to its pathogenesis: spontaneous and traumatic. Spontaneous pneumopericardium in neonates is associated with pulmonary diseases such as hypoplasia and respiratory distress syndrome [2]. Traumatic pneumopericardium occurs by pleural–pericardial communication associated with chest trauma and iatrogenic chest injury. Mechanism of pneumopericardium is presence of direct communication between the pericardium and airways. In addition, pericardium has relatively more negative pressure than intrapleural pressure [3]. Hence, in both spontaneous and traumatic pneumopericardium, PPV could be an exacerbating factor and should be avoided once pneumopericardium is diagnosed [4–6].

When clinically categorized, pneumopericardium is divided into nontension and tension. Tension pneumopericardium leads to hemodynamic collapse which should be treated immediately [7]. Pneumopericardium resulting in cardiac tamponade had been reported to receive PPV [1]. This is also the reason that PPV for patient with pneumopericardium is avoided.

Here, we present a 28-day-old neonate undergoing pericardiocentesis in sub-acute phase after aortic arch anastomosis who developed cardiac tamponade secondary to tension pneumopericardium under spontaneous breathing. Unlike usual pneumopericardium in neonates, the pneumopericardium in this patient exacerbated with increasing spontaneous inspiratory effort due to upper airway stenosis. We ceased spontaneous breathing and successfully treated the tension pneumopericardium by tracheal intubation and PPV.

## Case presentation

**Fig. 1:**
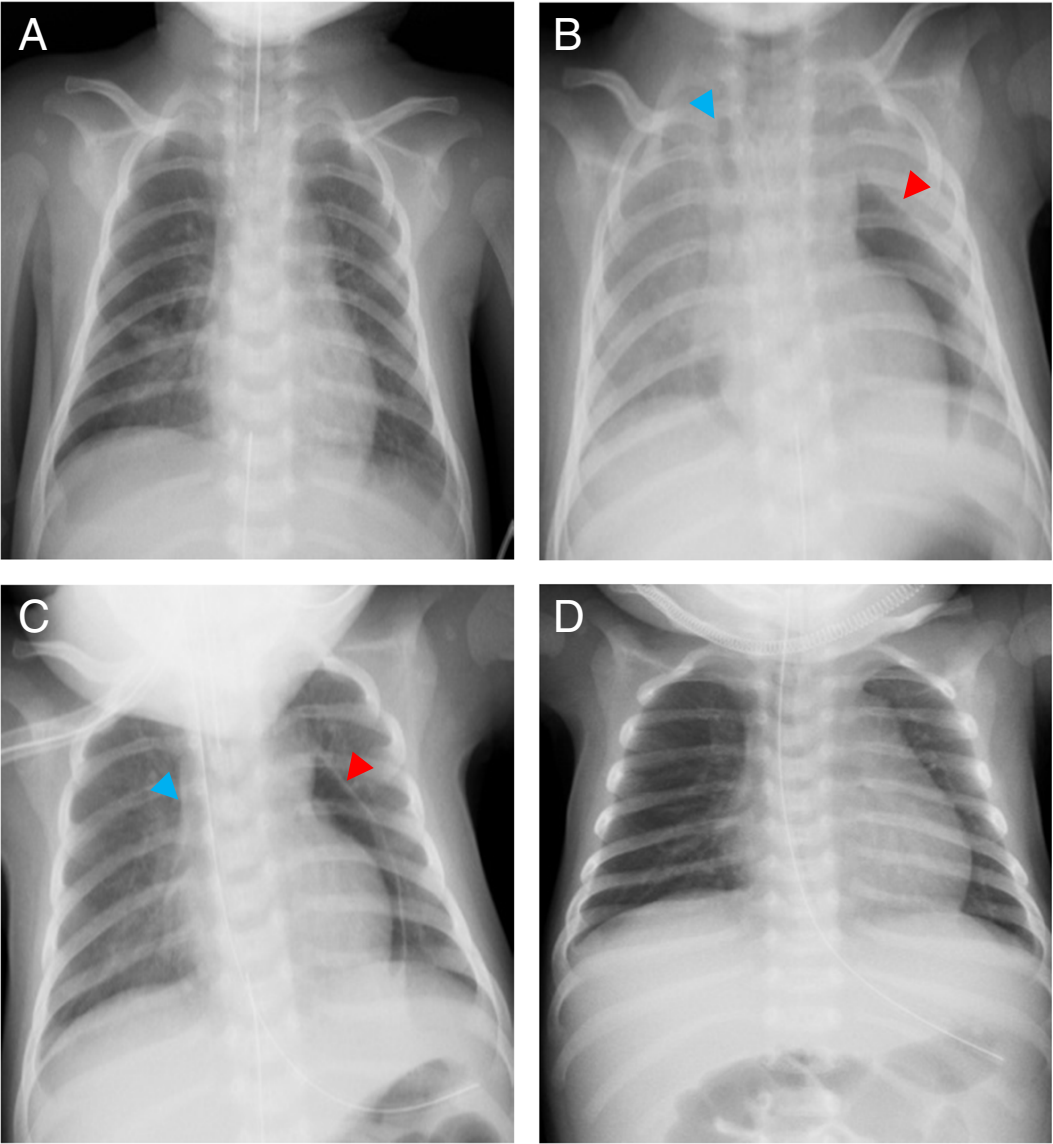
Chest X-rays in the operating room and in the intensive care unit. **a** Right after the pericardiocentesis, **b** before re-intubation approximately 2.5 h after admission to the ICU, **c** 10 min after re-intubation, and **d** after the drainage tube was removed. The red and blue arrows indicate pneumopericardium and pneumomediastinum, respectively

**Fig. 2:**
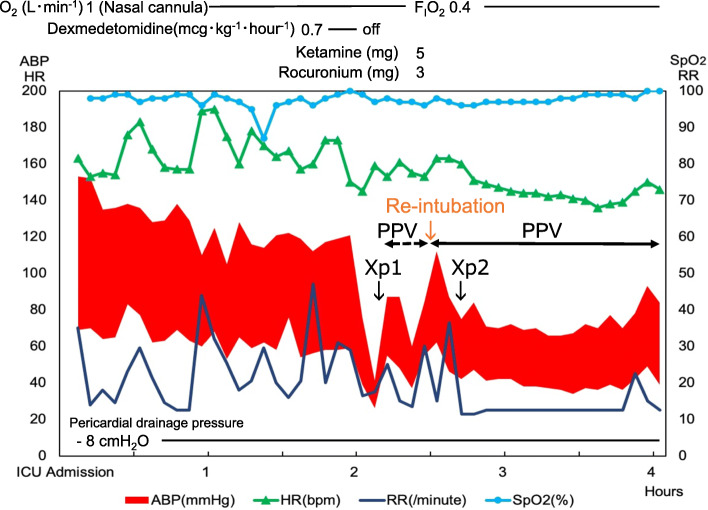
Clinical course in the intensive care unit. O_2_, oxygen; F_I_O_2_, fraction of inspiratory oxygen; ABP, arterial blood pressure; HR, heart rate; bpm, beats per minute; RR, respiratory rate; SpO_2_, peripheral oxygen saturation; PPV, positive pressure ventilation. The chest X-rays of Fig. 1 b and c were taken at the time shown as Xp1 and Xp2, respectively, in this chart

This patient was a 28-day-old female neonate born at gestational age of 39 weeks with no prenatal diagnosis. The neonate was diagnosed as having interrupted aortic arch (IAA) type B, patent ductus arteriosus (PDA), ventricular septal defect (VSD), and atrial septal defect. At 13-days old, this neonate underwent aortic arch reconstruction by extended aortic arch anastomosis, PDA ligation, and VSD patch closure under general anesthesia using 3.5 mm of uncuffed endotracheal tube (ETT). Immediately after the extubation on postoperative day (POD) 2, the patient presented hoarseness and severe stridor that were diagnosed as left recurrent nerve palsy by an otolaryngologist with fiberoptic examination of the vocal cords. Although stridor occurred when asleep, the patient was uneventfully discharged from the intensive care unit (ICU) on POD4 without any respiratory support. On POD 15, elective pericardiocentesis in the operating room (OR) was scheduled for increasing pericardial effusion. Pericardiocentesis was performed uneventfully by epigastric approach under general anesthesia with tracheal intubation. Ten milliliters of serosanguineous fluid was drained, and a silicon drainage tube was left in the pericardium. The tube was connected to a drainage system applying negative pressure of 8 cmH_2_O. After confirming no pneumothorax or pneumopericardium on chest X-ray (Fig. 1a), the patient was extubated in the OR and was transferred to the ICU. The clinical course of this patient after admission to the ICU is shown on Fig. 2.

In the ICU, the patient presented with tachypnea, tachycardia, hypertension, and stridor, and was given acetaminophen for analgesia. The acetaminophen was not effective, and the patient was administered 0.7 mcg ⋅ kg^−1^ ⋅ hour^−1^ of dexmedetomidine for analgesia and sedation in the hope of decreasing work of breathing. However, the stridor deteriorated, and retraction developed. In addition, systolic arterial blood pressure (sABP) dropped to 40 mmHg in 15 min although the heart rate was kept over 140 beats per minute. Because of new onset of mandibular breathing in addition to progressive hypotension, we stopped dexmedetomidine infusion and started bag-valve-mask ventilation 2 h after admission to the ICU. After starting positive pressure ventilation, blood pressure slightly increased. A chest X-ray revealed pneumopericardium and pneumomediastinum without pneumothorax (Fig. 1b). The cause of the hemodynamic deterioration was thought to be developing cardiac tamponade secondary to tension pneumopericardium. Inspection of the drainage system showed no loose connection which could suck air into the pericardium through the drainage tube or obstruction by blood, clots, and bending. In addition, we confirmed that excessive negative pleural pressure was generated because negative pressure alarm in the drainage system sounded and the drainage fluid was about to draw into the pericardial space. As a result, we concluded that (1) inspiratory effort by upper airway stenosis due to the existing left recurrent nerve palsy was exacerbated by glossoptosis induced by sedation and that (2) negative pleural pressure augmented by the increased inspiratory effort caused air suction into pericardium via insertion route of a drainage tube. The patient was intubated and was placed on mechanical ventilation. Eventually, sABP became stable to 70 mmHg, and pneumopericardium decreased on the chest X-ray (Fig. 1c). Two days later, confirming no remaining pneumopericardium or adverse events, the drainage tube was removed, and the patient was extubated again (Fig. 1d). The patient used high flow nasal cannula for 1 day in the ICU and spent an additional 6 days in the ward with no recurrent pericardial effusion or pneumopericardium before being discharged from the hospital.

## Discussion

This is a case in which pneumopericardium after pericardiocentesis developed by excessive negative pleural pressure due to upper airway stenosis. The cause of the pneumopericardium and the reason that PPV was effective are discussed below.

In this case, pneumopericardium resulting in cardiac tamponade was successfully treated by PPV. Cummings et al. reported that over 60% of causes of pneumopericardium leading to cardiac tamponade were chest trauma and diseases in lung-pleura [1]. And, PPV is one of the reasons for hemodynamic collapse. Thus, it was critical to diagnose the cause of the pneumopericardium in the patient to justify PPV for improving hemodynamic deterioration. This patient did not have any lung disease. In addition, neither pneumothorax nor pneumopericardium were confirmed in the chest X-ray during PPV in the OR. These findings suggested this pneumopericardium occurred by some causes which happened after extubation. Pneumopericardium after pericardiocentesis was reported to occur by pleural-pericardial communication [8–10]. However, no air in the pleural space or loose connection in the drainage was found. Absence of air in the pleural space with existence of pneumopericardium under negative pressure ventilation indicated that there was no pleural-pericardial communication. Eventually, we diagnosed that the cause of pneumopericardium was the negative pressure applied to the pericardium which exceeded drainage suction pressure and that air was sucked through the slit between the skin and the pericardium drainage tube.

Another distinctive feature in this case is the reason for excessive negative intra-pericardial pressure. First, increasing inspiratory effort by upper airway stenosis produced markedly negative pleural pressure. The patient had left recurrent nerve palsy as a complication after repair of aortic arch anastomosis for IAA (type B). It was reported that vocal cord palsy occurred in 47.2% of infants after aortic arch augmentation for IAA or hypoplastic aortic arch [11]. In addition to the recurrent nerve palsy, glossoptosis induced by sedation aggravated upper airway stenosis. The negative airway pressure elicited airway deformity, and triggered ventilatory overshoot [12], which exacerbated negative pleural pressure. Because the pericardium is contiguous with pleural space, the negative pleural pressure was propagated to the pericardium.

A previous case report presented pneumopericardium by leaky drainage system [10]. However, there was no loose connection in the pericardium drainage system, and - 8 cmH_2_O was applied to the system in this case. Negative airway pressure accompanied by obstructive upper airway has been reported to exceed - 50 cmH_2_O [13, 14]. The vacuum pressure of the drainage was much less than negative pleural pressure in this patient and was not effective to evacuate the air that was drawn from the atmosphere.

PPV had two effects in this case: (1) positive pressure was propagated to the pericardium and stopped the sucking of air into the pericardium from the slit between the skin and the drainage tube and (2) air in the pericardium was drained into the drainage bottle because the pericardial pressure was relatively positive compared with the pressure applied to the drainage bottle.

There is a limitation in our approach to this pneumopericardium. We diagnosed this patient as cardiac tamponade just by clinical symptoms and chest X-ray. We should have performed echocardiogram to confirm the diagnosis and to assess effectiveness of the therapeutic intervention.

In conclusion, positive airway pressure is not always an exacerbating factor of pneumopericardium. In a patient with pericardial-atmosphere communication, increased inspiration effort can be a cause of pneumopericardium, resulting in cardiac tamponade, and PPV is a therapeutic option to alleviate the pneumopericardium.

## Data Availability

Not applicable due to patient privacy concerns.

## Abbreviations

PPV: Positive pressure ventilation
IAA: Interrupted aortic arch
PDA: Patent ductus arteriosus
VSD: Ventricular septal defect
ETT: Endotracheal tube
POD: Postoperative day
ICU: Intensive care unit
OR: Operating room
sABP: Systolic arterial blood pressure
O_2_: Oxygen
F_I_O_2_: Fraction of inspiratory oxygen
ABP: Arterial blood pressure
HR: Heart rate
bpm: Beats per minute
RR: Respiratory rate
SpO_2_: Peripheral oxygen saturation
